# Retraction of: miR-34a inhibits melanoma growth by targeting ZEB1

**DOI:** 10.18632/aging.206155

**Published:** 2024-11-30

**Authors:** Yazhen Xu, Bingyu Guo, Xiaoyan Liu, Kai Tao

**Affiliations:** 1 Reconstructive and Plastic Surgery, General Hospital of Northern Theater Command, Shenyang, P.R. China

**Keywords:** miR-34a, melanoma, ZEB1, proliferation, migration

**This article has been retracted:** Aging has completed its investigation of this paper. We found multiple instances of overlap between transwell assay images in this article and images from previously published unrelated papers. Specifically, the transwell assay images in Figure 2G, representing cell migration data, overlap images from unrelated papers [1–4] and the image in Figure 2H overlaps an image from retracted paper [5]. Also, the transwell assay images in Figures 2H and 2I overlap images from earlier published papers [6, 7]. In addition, the ZEB1 western blot panel in Figure 3E is a duplication of the GAPDH panel in [8]. The Scientific Integrity office at Aging contacted the authors and they admitted “improper data storage” and “repeated use of images in the article.” Consequently, all authors agreed that the article should be retracted.
